# KDM6 Demethylases and Their Roles in Human Cancers

**DOI:** 10.3389/fonc.2021.779918

**Published:** 2021-12-07

**Authors:** Chunyan Hua, Jiaqing Chen, Shuting Li, Jianan Zhou, Jiahong Fu, Weijian Sun, Wenqian Wang

**Affiliations:** ^1^ School of Basic Medical Sciences, Wenzhou Medical University, Wenzhou, China; ^2^ Wenzhou Medical University, Wenzhou, China; ^3^ Department of Surgery, The Second Affiliated Hospital and Yuying Children’s Hospital of Wenzhou Medical University, Wenzhou, China

**Keywords:** histone demethylase, KDM6, cancer, epigenetics, cancer therapy

## Abstract

Cancer therapy is moving beyond traditional chemotherapy to include epigenetic approaches. KDM6 demethylases are dynamic regulation of gene expression by histone demethylation in response to diverse stimuli, and thus their dysregulation has been observed in various cancers. In this review, we first briefly introduce structural features of KDM6 subfamily, and then discuss the regulation of KDM6, which involves the coordinated control between cellular metabolism (intrinsic regulators) and tumor microenvironment (extrinsic stimuli). We further describe the aberrant functions of KDM6 in human cancers, acting as either a tumor suppressor or an oncoprotein in a context-dependent manner. Finally, we propose potential therapy of KDM6 enzymes based on their structural features, epigenetics, and immunomodulatory mechanisms, providing novel insights for prevention and treatment of cancers.

## Introduction

The initiation and progression of cancer, traditionally viewed as a set of genetic diseases, have now been considered as a complex cooperation within genetic alterations and epigenetic abnormalities (1). Epigenetics including DNA methylation, histone modification, and chromatin remodeling is involved in the regulation of gene expression and is closely related to multiple human cancers (2). Histone methylation and demethylation play an important role in histone modification (3). The histone H3 lysine 27 (H3K27) methylation status is dominated by histone methyltransferase EZH2 and two lysine demethylases (KDMs), and deregulated H3K27 methylation is often associated with a multitude of cancer types (4, 5).

KDM6 enzymes are capable of removing di-methylated and tri-methylated H3K27, thereby activating or repressing target gene transcription (6). Notably, KDM6 demethylases appear to be highly regulated at the transcriptional level and more susceptible to diverse stimuli like tumor microenvironments, metabolic reactions, differentiation inducers and stress signals (7, 8). There are emerging evidences for deregulation of KDM6 demethylases and important phenotypic consequences in various types of cancer (9–11). However, the precise molecular basis of how these lysine-specific demethylases contribute to oncogenesis has not been extensively pursued. This review summarizes recent advances in understanding structure, alterations and functions of KDM6 histone demethylases in oncogenesis and potential therapeutic targeting of these enzymes, providing a train of thought for the prevention and treatment of cancers.

## Structural Features of KDM6 Enzymes

**Figure 1:**
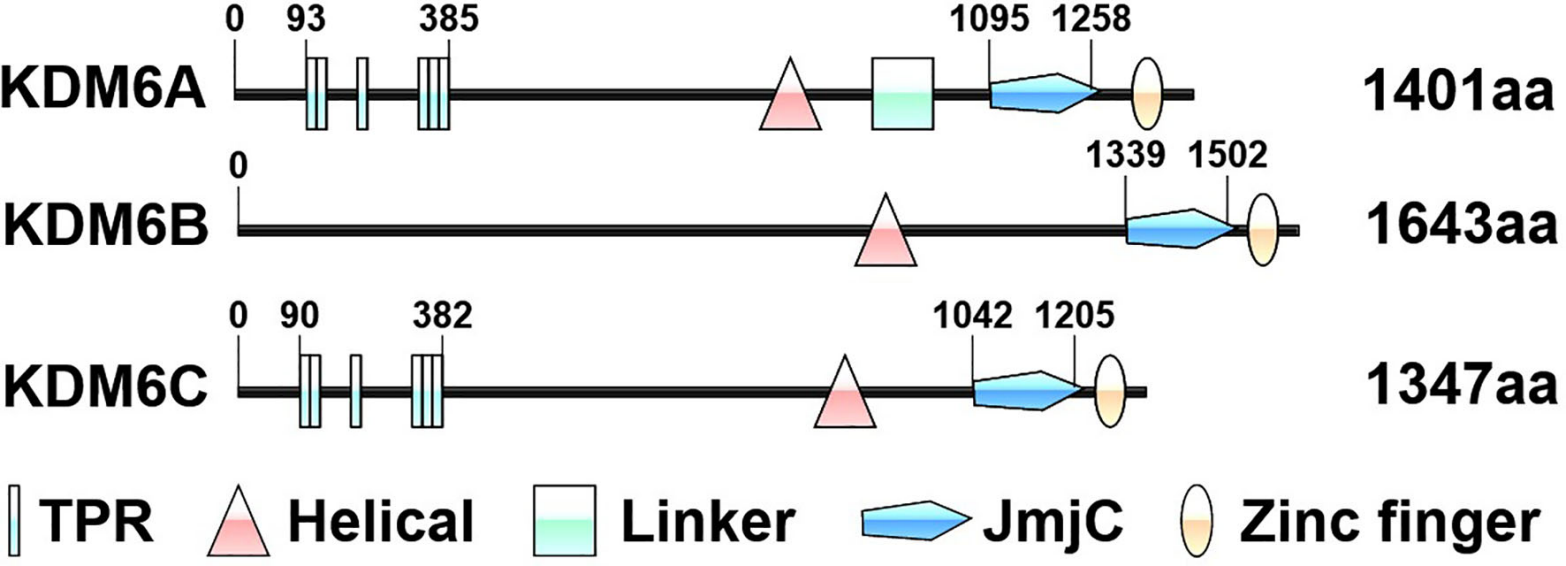
Schematic representation of the protein domains of KDM6 enzymes. The structure of KDM6 members consists of five regions: tetratricopeptide repeat domain (TPR), helical domain, linker region, Jumonji C (JmjC) domain and zinc finger domain. The numbers indicate the amino acid residues. The domains are based on the UniProt database (https://www.uniprot.org/).

The KDM6 demethylase subfamily belongs to the Jumonji C (JmjC) domain-containing KDMs, these enzymes require ferrous iron Fe(II), α-ketoglutarate [α-KG, also known as 2-oxoglutarate (2-OG)] and oxygen as co-factors (12). Due to these dependencies, KDM6 demethylases are more responsive to tumor microenvironments and cellular metabolites (10). KDM6 subfamily consists of three distinct members called KDM6A (also known as UTX), KDM6B (also known as JMJD3) and KDM6C (also known as UTY) proteins (13). Whereas KDM6A and KDM6C are respectively located at X and Y chromosomes, KDM6B is found on chromosome 17 (13). The structure of KDM6 members consists of five regions: tetratricopeptide repeat domain (TPR), helical domain, linker region, JmjC domain and a GATA-like zinc finger domain (**Figure 1**) (12). All three KDM6 members contain JmjC domains at their C termini catalyzing protein lysine demethylation, especially H3K27 dimethylation (H3K27me2) and H3K27 trimethylation (H3K27me3) (10). Of note, demethylase activity of KDM6C is minimal because of a subtle sequence divergence in the JmjC catalytic domain (10). However, in a knock-out mouse model, KDM6C can partially compensate for the demethylase-independent functions of KDM6A (14). The zinc finger domain of KDM6 subfamily is found to have a DNA binding function and plays an important role in the regulatory regions of genes (12, 15). KDM6A and KDM6C, but not KDM6B, have similar N-terminal TPR domains to form scaffold which could mediate protein-protein interactions and the assembly of multiprotein complexes (10). The helical domain of KDMs is anticipated to play roles in specific protein-protein interactions both in the assembly of chromatin-protein and other protein complexes (16). However, to date, the role of the helical domain in the KDM6 subfamily remains unclear, as the amine oxidase like domain serves the active site for substrate and cofactor binding. Whereas enzymatic roles of KDM6A and KDM6B are undisputed in cancer cells, non-enzymatic scaffolding roles for large complexes of all KDM6 members have been recognized (17). In a growing number of cases, mutations affecting these KDM6 response to specific therapies (18, 19).

## The Regulation of KDM6 Signaling

**Figure 2:**
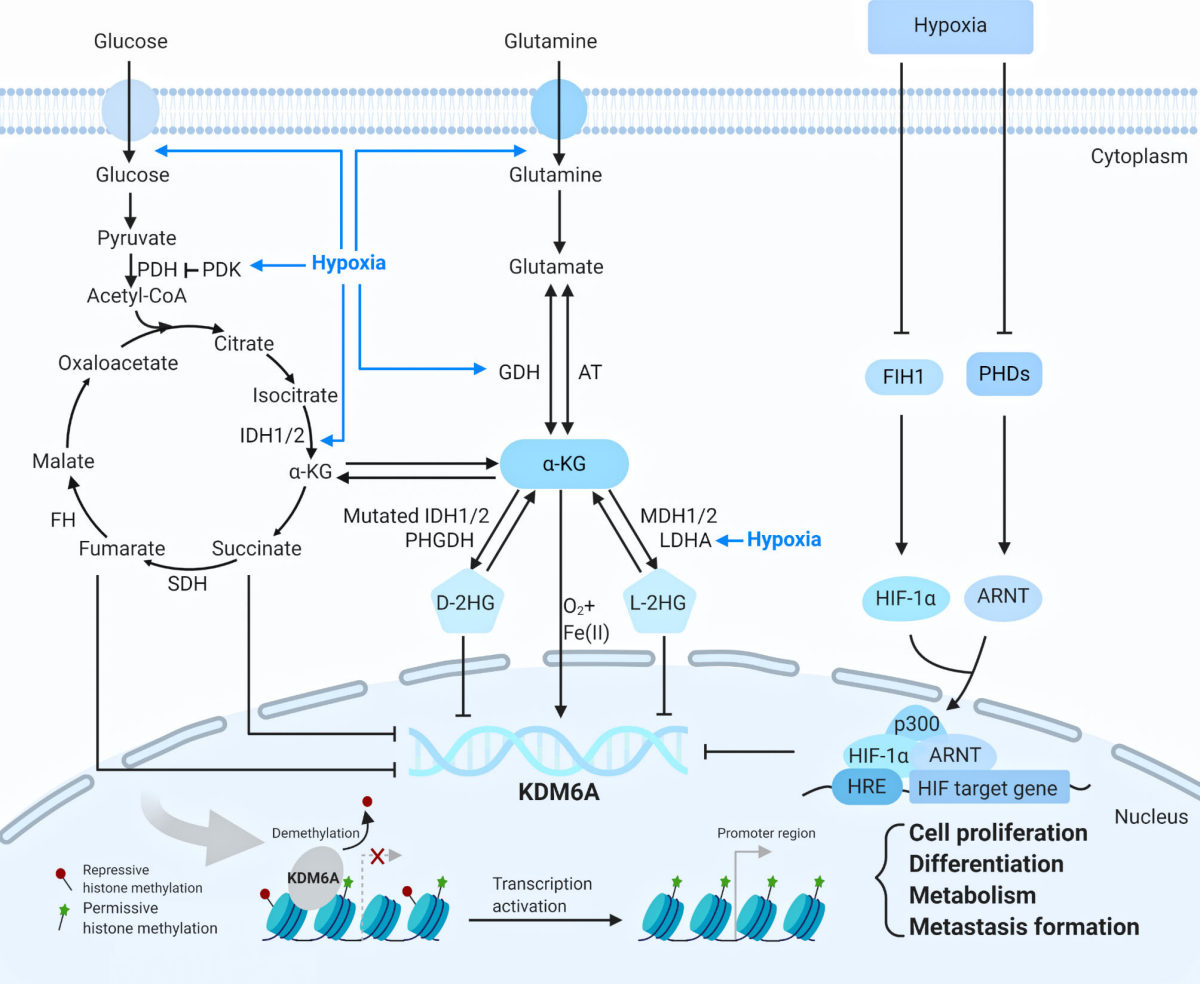
The regulation of KDM6A (Created with BioRender.com). KDM6A is upregulated or downregulated by metabolic reactions and hypoxia through diverse mechanisms. KDM6A is re-cruited to the chromatin and respond to the transcription through influencing cell proliferation, differentiation, metabolism and metastasis formation. ARNT, aryl hydrocarbon nuclear translocator, AT amino transferases, D-2HG D-2-hydroxyglutarate, FH fumarate hydratase, FIH1 factor-inhibiting hypoxia-inducible factor 1, GDH glutamate dehydrogenases, HIF-1α hypoxia-inducible factor-1α, HRE hypoxia response element, IDH1/2 isocitrate dehydrogenases 1 or 2, LDHA lactate dehydrogenase A, L-2HG L-2- hy-droxyglutarate, MDH 1/2 malate dehydrogenase 1 or 2, PDH pyruvate dehydrogenase, PHDs prolyl hydroxylases, PDK pyruvate dehydrogenase kinase, PHGDH D-3-phosphoglycerate, SDH succinate dehydrogenase, α-KG α-ketoglutarate.

Given that KDM6 demethylases are involved in the regulation of a vast array of biological processes *via* their demethylase dependent or independent functions, their levels and associated activities have to be precisely controlled. However, this area of research has never been addressed. Below is the summary of available reports on the regulation of KDM6 (**Figure 2**).

KDM6A is regulated by metabolic reactions and hypoxia through diverse mechanisms. As KDM6A uses cellular metabolites such as Fe(II), and tricarboxylic acid (TCA) cycle intermediate α-KG and oxygen for their catalytic reactions, its activity is influenced by mutations in the TCA cycle enzymes isocitrate dehydrogenase 1 and 2 (IDH1 and 2), succinate dehydrogenase (SDH), and fumarase hydratase (FH). Glucose and glutamine respectively are transported into cells and both are converted to α-KG, which related to 2-hydroxyglutarate (2-HG) (10). The metabolite 2-HG can be produced as either a D(R)- or L(S)-enantiomer, each of which functions as a potent inhibitor of KDM6A enzyme involved in diverse biologic processes (20). Oncogenic mutations in IDH1 or 2 produce D-2HG, which causes a pathologic blockade in cell differentiation (21). Production of L-2HG instead results from promiscuous substrate usage primarily by lactate dehydrogenase A (LDHA) (**Figure 2**) (20). Hypoxic tumor microenvironment is closely associated with cellular metabolites (10). Under hypoxia, effective concentration of α-KG is decreased, and the 2-HG level is increased *via* metabolic reprogramming, thus contributing to the inhibition of α-KG-dependent KDM6A, which is related to cellular heterogeneity, cancer resistance, and progression (20). Interestingly, in the latest study KDM6A was found to be a direct sensor of oxygen (22, 23). In hypoxic conditions, factor-inhibiting hypoxia-inducible factor 1 (FIH1) and prolyl hydroxylases (PHDs) are inactivated, allowing hypoxia-inducible factor-1α (HIF-1α) to dimerize with aryl hydrocarbon nuclear translocator (ARNT; also called HIF-1β) (24). The HIF complex translocates to the nucleus and binds to hypoxia response element (HRE) in HIF target gene promoters (24). KDM6A inactivation *via* hypoxia promotes hypermethylation of H3K27, preventing transcriptional reprogramming for cellular differentiation (**Figure 2**) (22, 24).

KDM6A also exerted the function under the stimulation of certain specific proteins and ligands. Recent studies have demonstrated demethylase-independent functions of KDM6A as a scaffold protein that facilitates the binding of other factors that directly regulate transcription. The KDM6A were also found to be associated with estrogen receptor (ER) and retinoic acid receptor (RAR) upon ligand treatments, including estrogens and retinoic acid, which is necessary for cellular differentiation, cancer tumorigenesis and metastasis (13). Although whether KDM6A interacts with ER or RAR directly, or depends on certain complex, remains unclear, these studies have shed light that KDM6A contributes to oncogenesis on the ligand-dependent transcriptional regulation (25, 26).

**Figure 3:**
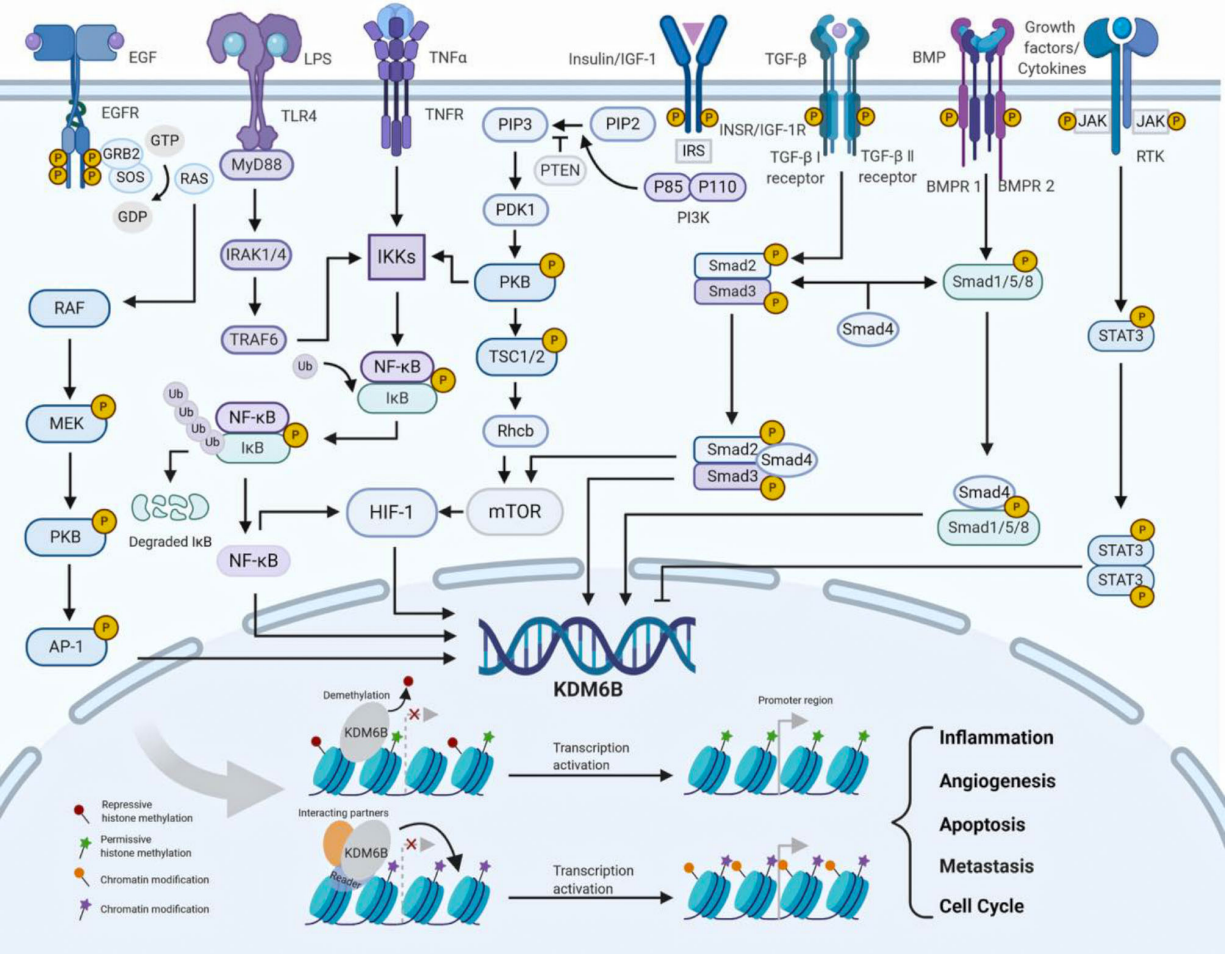
The regulation of KDM6B (Created with BioRender.com). KDM6B is upregulated or downregulated by growth factors, cytokines and cellular stresses via distinct signaling pathways. KDM6B is recruited to the chromatin and involved in a wide range of biological processes, such as angiogenesis, metastasis, cell cycle, apoptosis and inflammation. AP-1 activator protein 1, BMP bone morphogenetic protein, BMPR1 bone morphogenetic protein receptor 1, BMPR2 bone morphogenetic protein receptor 2, EGF epidermal growth factor, EGFR epidermal growth factor receptor, ERK extracellular signal-regulated kinase, GRB2 growth factor receptor bound protein 2, HIF-1 hypoxia inducible factor-1, IGF-1 insulin-like growth factor 1, IGF-1R insulin-like growth factor 1 receptor, IκB inhibitor of nuclear factor-κB, IKKs inhibitor of nuclear factor-κ kinases, INSR insulin receptor, IRAK1/4 interleukin-1 receptor-associated kinase 1/4, IRS insulin receptor substrate, JAK Janus kinase, LPS lipopolysaccharide, MEK MAPK/ERK kinase, MAPK mitogen-activated protein kinase, mTOR mammalian target of rapamycin, MyD88 myeloiddifferentiationfactor88, NF-κB nuclear factor-κB, PI3K phosphoinositide 3-kinase, PIP2 phosphatidylinositol- 3,4-bisphosphate, PIP3 phosphatidylinositol-3,4,5-trisphosphate, PDK1 3-phosphoinositide-dependent protein kinase 1, PTEN phosphatase and tensin homolog deleted on chromosome ten, PKB protein kinase B, RTK receptor tyrosine kinase, SOS son of sevenless, STAT3 signal transducer and activator of transcrip-tion 3 , TGF-β transforming growth factor-β, TLR4 toll-like receptor 4, TNF-α tumor necrosis factor-α, TNFR tumor necrosis factor receptor, TRAF6 TNF receptor-associated factor 6, TSC1/2 tuberous sclerosis complex 1/2.

The role of KDM6B in cell proliferation and differentiation is regulated by growth factors, cytokines and ligands *via* distinct signaling pathways in human cancers. KDM6B is upregulated *via* nuclear factor-κB (NF-κB) binding to its promotor region by tumor necrosis factor α (TNFα), and that KDM6B in turn upregulates mitogen-activated protein kinase (MAPK) pathway, thereby promoting cancer cell growth and survival in a catalytically-independent manner (**Figure 3**) (27, 28). RAS is activated by GDP/GTP exchange stimulated by epidermal growth factor (EGF), and activator protein 1 (AP-1) transcription factors are good candidates for acting as transactivators of KDM6B transcription (29). KDM6B expression is induced by activation of RAS–RAF pathway, and contributes to activate tumor suppressor proteins p16INK4A and p14ARF, which triggers cellular senescence and apoptosis in a demethylase-dependent fashion (**Figure 3**) (30, 31). Signal transducer and activator of transcription 3 (STAT3) is activated by phosphorylation on receptor-associated Janus kinases (JAKs), and regulates the proliferation and self-renewal ability of cancer stem cells through repression of KDM6B expression (**Figure 3**) (32). Moreover, like KDM6A, KDM6B interacts with ER or RAR in the presence of estrogens or retinoic acid, and regulates the proliferation and differentiation of cancer cells. However, the mechanisms underlying KDM6B interaction with ER or RAR are unknown (33).

KDM6B is upregulated or downregulated by different signaling pathways, and play essential roles in the tumor microenvironment. It has been previously shown that bone morphogenetic protein 4 (BMP4) activates the Smad signaling cascade and upregulates KDM6B expression as the regulator of balances between self-renewal and differentiation. Interestingly, a positive feedback loop is found between KDM6B and BMP signaling, and KDM6B also could employs BMP and NF-kB pathways to modulate the tumor microenvironment by angiogenesis and tumor associated-macrophage infiltration (**Figure 3**) (34, 35). Recently, the mechanisms of KDM6B in regulating inflammatory genes have been studied extensively and deeply, supporting the view that KDM6B involves the engagement of an inflammatory tumor microenvironment (36). TNF receptor associated factor 6 (TRAF6) is activated by toll like receptor 4 (TLR4) signaling stimulated by lipopolysaccharides (LPS), and induces phosphorylation of the inhibitor of nuclear factor-κ kinase (IKK) complex, leading to activation of KDM6B *via* NF-κB pathway (28, 37). Consequently, KDM6B is recruited to promoter regions of inflammatory genes TNF-α and interleukin-6 (IL-6), where the H3K27me3 levels strikingly decrease (**Figure 3**) (38, 39). KDM6B is upregulated *via* Smad signaling by activated by transforming growth factor-β (TGF-β), and that KDM6B in turn upregulates SNAI1, which is a master transcription factor in epithelial-mesenchymal transition (EMT) and cancer progression (**Figure 3**) (40, 41).

The regulation of KDM6B is an important host response against cellular metabolism and environmental stress. KDM6B expression is up-regulated through HIF-1 under hypoxia and normoxia (29). Independently from oxygen levels, HIF-1 is constitutively transcribed and synthesized through a series of signaling events involving IGF-1/PI3K/mTOR, TNF-α/NF-κB and TGF-β/Smad/mTOR pathways (**Figure 3**) (42–46). Moreover, similar to KDM6A, KDM6B expression is also subjected to different levels of its cosubstrates from cellular metabolism and tumor microenvironments (10). Studies have demonstrated vitamin D metabolite 1α,25-dihydroxyvitamin D (3) [1,25(OH) (2)D (3)] modulates the KDM6B gene promoter and increases the level of KDM6B RNA in human colon cancer cells (47). Regional glutamine deficiency decreases KDM6B expression, which promotes tumor heterogeneity and therapeutic resistance (48). However, the functions of KDM6B in cellular metabolism as well as regulatory mechanisms of above-mentioned stresses remain to be fully studied.

KDM6C is an essential downstream mediator of NKX3.1 for prostate differentiation and cancer (49). Compared with other two paralogs, very little is known as to how KDM6C is regulated under physiological and pathological conditions. Thus, elucidation of these biological processes may help to understand the context dependent role of KDM6C in human cancers.

## KDM6 in Cancer

KDM6 profoundly influences gene expression, based on enzymatic activity in demethylating H3K27me3, and non-enzymatic scaffolding roles for large complexes that open and close chromatin for transcription. KDM6 plays a crucial and dual role in onset and progression of cancers through activating or repressing target gene transcription, which has been recognized to strongly influence cancer risk, prognosis, and therapy resistance.

In recent years, KDM6A is found to be aberrantly expressed in many cancers as a tumor suppressor or promoter, implying its regulatory role in tumor initiation and progression (**Table 1**). KDM6A is initially a tumor-suppressor of Notch and Rb-dependent tumors in Drosophila (117). Numerous studies have demonstrated that KDM6A is targeted by loss-of-function mutations as a suppressor in various cancers, including acute myeloid leukemia, B cell lymphoma, multiple myeloma, non-small cell lung cancer, squamous-like pancreatic cancer, T-cell acute lymphoblastic leukemia, urothelial cancer, colorectal cancer, medulloblastoma, breast cancer, hepatocellular cancer and esophageal squamous cell cancer (50–58, 60, 61, 67–73, 118).

**Table 1:** Histone substrates, expression levels, effects and target genes of KDM6 in various cancers.

Histone Demethylase	Histone Substrates	Expression Level	Cancer Type	Effect	Target Gene	Refs
KDM6A	H3K27me2 H3K27me3	Loss	Acute Myeloid Leukemia	Suppressor	ETS、GATA、ENT1	(50–52)
			B Cell Lymphoma	Suppressor	Efnb1	(53)
			Multiple Myeloma	Suppressor	NCAM1、AOC3、CDHR5、 E-cadherin	(54, 55)
			Non-Small Cell Lung Cancer	Suppressor	KRAS、E-cadherin	(56, 57)
			Squamous-like Pancreatic Cancer	Suppressor Promoter	ΔNp63, MYC, RUNX3 TP63∆N	(58, 59)
			T-cell Acute Lymphoblastic Leukemia	Suppressor	NOTCH1	(60)
			Urothelial Cancer	Suppressor	FGFR3、PIK3CA、P53、 KMT2C/D	(59, 61–66)
			Colorectal Cancer	Suppressor	E-cadherin	(67, 68)
			Medulloblastoma	Suppressor	Cxcl9、Cxcl13、Ccl21a	(69)
			Breast Cancer	Suppressor	E-cadherin、Dicer	(70, 71)
			Hepatocellular Cancer	Suppressor	Smad2、E-cadherin	(72, 73)
		High	T-cell Acute Lymphoblastic Leukemia	Promoter	TAL1	(74)
			Retinoblastoma	Suppressor	Rb、Rbl2	(75)
			Breast Cancer	Promoter	ERα、CXCR4、OCT4	(25, 76)
			Prostate Cancer	Promoter	AR、Wnt/β	(77–79)
			Cervical Cancer	Promoter	p21^CIP1^	(80)
			Acute Myeloid Leukemia	Promoter	DOCK5/8	(81)
			Glioblastoma	Suppressor	MGMT	(82)
				Promoter	NOTCH	(83)
			Non-Small Cell Lung Cancer	Promoter	KMT2B	(84)
			Adenoid Cystic Cancer	Promoter	NOTCH	(85)
			Melanoma	Suppressor	MYC、IFN	(86)
			Ovarian Cancer	Promoter	CD44、NANOG、c-MYC	(87)
KDM6B	H3K27me2 H3K27me3	Low	High Risk Neuroblastoma	Suppressor	NEFM	(88)
			Colorectal Cancer	Suppressor	p15^INK4B^	(89)
			Acute Myeloid Leukemia	Suppressor	*C/*EBPβ、RIPK3	(90)
			Pancreatic Cancer	Suppressor	C/EBPα	(91)
			Squamous Cell Cancer	Suppressor	CCNB1、CDK1、IL6	(92)
		High	Acute Myeloid Leukemia	Promoter	HOX、AP1	(80, 93, 94)
			Cervical Cancer	Promoter	p16^INK4A^	(95)
			Multiple Myeloma	Promoter	ELK1、FOS	(27)
			Diffuse Large B-Cell Lymphoma	Promoter	IRF4	(96)
			Prostate Cancer Gastric Cancer	Promoter Promoter	PTEN、cyclin D1 TP53	(97–99)
			Hepatocellular cancer	Promoter	SLUG、TIAM1	(100, 101)
			Ovarian Cancer	Promoter	TGF-β1、HER2	(102, 103)
			T-cell Acute Lymphoblastic Leukemia	Promoter	HES1、AP1、NOTCH1	(27, 94, 104)
			Breast Cancer	Promoter	OCT4、NANOG、SOX2、BCL2、IGFBP5	(105–107)
			Hodgkin’s Lymphoma	Promoter	CD58、NOTCH2NL	(108)
			Colorectal Cancer	Promoter	CXCL9、CXCL10	(109)
			Melanoma	Promoter	STC1、CCL2	(110, 111)
			Non-small Cell Lung Cancer	Promoter	E-cadherin	(112, 113)
			Chronic Myelomonocytic Leukemia	Promoter	S100A9、TLR、C3	(114)
			Renal Cell Cancer	Promoter	SNAI1	(115)
			Chordoma	Promoter	TBXT	(116)
			Glioblastoma	Promoter	NOTCH	(83)
KDM6C	Unknown	Loss	Acute Myeloid Leukemia	Suppressor	ETS	(52)
			Squamous-like Pancreatic Cancer	Suppressor	P63、MYC	(58)

KDM6A mutations are commonly lost in all stages and subtypes of urothelial cancer (UC), which is classified into non-muscle-invasive and muscle-invasive tumors (62–65). Papillary UC is driven by loss of mutation of KDM6A in growth signaling pathway, especially activating FGFR3 and PIK3CA (119). In muscle-invasive UC, KDM6A deficiency activates multiple immune response genes and causes bladder cancer in cooperation with p53 dysfunction (120). In the TCGA analysis of muscle-invasive UC, KDM6A is found to interact with KMT2C and KMT2D in the compass complex (121, 122). Gender bias in KDM6A inactivation has been reported in some cancer types (58, 60, 66). More KDM6A mutations are present in non-muscle-invasive UC from females than males (123). Molecular mechanisms through which KDM6A suppresses the progression of urothelial cancer depend on the subtype, not only *via* its H3K27me3 demethylase activity but also *via* interactions with other epigenetic complexes.

The function of KDM6A is even different among subtypes of one disease, including acute myeloid leukemia, non-small cell lung cancer, squamous-like pancreatic cancer, T-cell acute lymphoblastic leukemia (T-ALL), breast cancer and glioblastoma (25, 50–52, 57–60, 68, 70, 71, 74, 76, 81–84). In human T-ALL, KDM6A acts as a tumor suppressor and is frequently genetically inactivated in a NOTCH1-induced T-ALL model (57). Interestingly, KDM6A controls oncogenic transcription factor TAL1 and functions as an oncogene for leukemia maintenance in human T-ALL (74). Yu and Taube et al. demonstrate that low expression of KDM6A predicts poor survival in breast cancer (70, 71). while Kim et al. show that high expression of KDM6A is associated with poor prognosis (25, 76). In breast cancer, KDM6A is found to be an important factor in mediating EMT (70, 71). KDM6A promotes the proliferation and migration of hormonally responsive breast cancer *via* feed-forward transcription with Erα (25). The dual roles of KDM6A suggest its context-specific role in tumorigenesis.

KDM6A also has been shown to be overexpressed and exert different roles in multiple human cancers (25, 74, 75, 77, 78, 80). The overexpression of KDM6A changes histone H3 methylation on the promoters of tumor suppressor gene Rb and Rbl2, contributing to the decreased cell proliferation of retinoblastoma (75). Analysis of TCGA database found that high KDM6A is associated with sex-biased by activating immune-related pathways (86). Nevertheless, KDM6A cannot be perceived solely as a tumor suppressor, as KDM6A is found to have close association with genesis and progression of different cancers (25, 74, 77, 78, 80). In advanced stages of prostate cancer, KDM6A is essential for maintenance of androgen receptor (AR) activity despite anti-androgenic therapy (77, 78, 124). A different pro-oncogenic function has been shown in cervical cancer, where increased KDM6A expression appears to support cell cycle activation by p21CIP1 suppression of replication stress (80). KDM6A and NOTCH1 mutations are assocatiated with poor prognosis in metastatic adenoid cystic cancer (85). KDM6A interacting with GATA activates stem-like phenotypes in ovarian cancer cell lines (87).

KDM6B mediates carcinogenic and anti-cancer signaling pathways by directing distinct transcription factors in a context-dependent manner, imposing wide-ranging effects on proliferation, apoptosis, migration, stem cell behavior, EMT, drug resistance and tumor microenvironment (**Table 1**). In several cancers, KDM6B is downregulated and regarded as a tumor suppressor by counteracting different transcriptional programs. KDM6B is downregulated in high-risk neuroblastomas, and functions as a tumor suppressor through neuronal differentiation by activating NEFM (88). Low expression of KDM6B is an independent predictor of poor prognosis in colorectal cancer by mediating p15INK4B expression (89). KDM6B expressional reduction is found in acute myeloid leukemia, and KDM6B acts as an oncorepressor by activating C/EBPβ-centered transcriptional program (90). Decreased expression of KDM6B enhances aggressiveness of pancreatic cancer through downregulation of C/EBPα (91). KDM6B shows its tumor-suppressive function on squamous cell carcinoma by mediating cell-cycle and proinflammatory genes, such as CCNB1, CDK1, and IL-6 (92).

However, KDM6B is more frequent upregulated and functions as tumor promotor in various cancers. KDM6B upregulates gene expression of cell cycle and proliferation related genes such as HOX, p16INK4A, ELK1, FOS, cyclin D1 and TP53, thereby promoting cell growth and survival of acute myeloid leukemia, cervical cancer, multiple myeloma, prostate cancer, and gastric cancer (27, 93, 95, 97, 99, 125). Overexpression of KDM6B exerts an anti-apoptotic effect in diffuse large B-cell lymphoma and prostate cancer, and respectively activates the transcription of downstream target genes IRF4 and PTEN *via* distinct mechanisms (75, 96, 98). High expression of KDM6B is correlated with distant metastasis *via* modulating H3K27me3 of SLUG gene promoter in hepatocellular carcinomas (100). KDM6B could promote the invasion of hepatocellular cancer, non-small cell lung cancer, renal cell cancer and chordoma through upregulation of EMT-specific genes, such as TIAM1, E-cadherin, SNAI1 and TBXT (101, 112, 113, 115, 116). Breast cancer cells acquire drug resistance to PI3K inhibitor *via* upregulation of KDM6B-mediated IGFBP5 expression (105). It has been reported that KDM6B impacts migration and invasion of ovarian cancer by regulating TGF-β1 and HER2 (102, 103). KDM6B regulates the differentiation of cancer stem cells *via* activating distinct target genes, and may contribute to the pathogenesis of T-ALL, acute myeloid leukemia, breast cancer, Hodgkin’s Lymphoma and Glioblastoma (27, 83, 94, 104, 106–108). In colorectal cancer and melanoma, KDM6B expression is elevated and positively correlated with several targets of CXCL9, CXCL10, STC1 and CCL2, which affects the tumor immune microenvironment by T cells and macrophage infiltration (109–111). In chronic myelomonocytic leukemia, KDM6B overexpression results in activation of innate immune genes, such as S100A9, TLR and C3, indicating that KDM6B is involved in tumor immune microenvironment (114).

Due to point substitutions affecting substrate binding, KDM6C, the Y-chromosome homolog of KDM6A, has markedly lower demethylase activity. Some studies confirm the frequent co-occurrence of KDM6A and KDM6C, and identify KDM6C as a demethylase independent tumor suppressor in acute myeloid leukemia and squamous-like pancreatic carcinoma (52, 58). Compared with other KDM6 subfamily, the function of KDM6C in human cancers needs further investigated.

The diversity of interacting transcription factors explains why KDM6 acts as a pro-oncogenic or tumor-suppressive factor in different tumor types. Notably, functional roles of KDM6 subfamily in tumorigenesis were found to be highly cell type-specific and pathologic context-dependent. Thus, taking into consideration the dual roles of KDM6 in different cellular contexts would make a better approach in overcoming tumor progression. In the same type of cancer, differential roles and associations of KDM6 subfamily in cancer should be further discussed in the future.

## KDM6 and Cancer Therapy

Since KDM6 demethylases are overexpressed in many cancers and contribute to cancer development, KDM6 family members have attracted considerable interest as targets for enzymatic inhibition. Most KDM6 demethylase inhibitors target the active site of JmjC domain *via* binding competitively with α-KG and chelating Fe(II) residue (126). Unfortunately, these agents are broad-spectrum inhibitors and also effect on other members of JmjC domain-containing family. Due to this reason, use of these compounds might result in significant cytotoxicity (127). Recently, some compounds inhibit a more restrictive subset of KDMs, with KDM6A being among the targets. MC3324, a dual KDM1A-KDM6A inhibitor, regulates drug resistance in ERα-positive breast cancer (128). Metformin has also been recognized as a catalytic inhibitor of KDM6A, and is currently in phase II clinical trials of bladder cancer (NCT03379909) (**Table 2**) (129). To date, the most described selective inhibitors of KDM6A and KDM6B are GSK-J1 and cell-active ethyl ester prodrug GSK-J4 (141). The synergistic inhibition of KDM6B and KDM1A by GSK-J1 and TCP is efficient in head and neck squamous cell carcinoma *in vivo* (130). GSK-J4 are relatively unspecific as it also inhibits KDM5 subfamily *in vitro* (142). GSK-J4 has nevertheless been proposed for application in several cancer types, particularly *in vivo* of acute myeloid leukemia, breast cancer, TAL1 positive T-ALL, colorectal cancer and osteosarcoma (**Table 2**) (74, 78, 93, 99, 106, 131–137). These indicate that GSK-J4 might be a promising treatment for clinical cancer therapy. The main obstacle of this treatment is achieving specific inhibition of key demethylases thus reducing possible toxicity or side effects. Inhibition of KDM6A and KDM6B might provide benefit in certain circumstances, but inhibition might produce deleterious responses. Based on intensive studies across a broad spectrum of tumors, the functional roles of KDM6A and KDM6B were inconsistent in the same type of cancer. If ablation of KDM6A or KDM6B increases cancer cell metastasis, GSK-J4 treatment might result in increased tumor growth instead. Therefore, development of highly specific small-molecule inhibitors of KDM6 subfamily will provide novel targeted therapeutic options. In addition, targeting non-catalytic functions of KDM6 will require chemical probes and inhibitors targeting protein-protein interactions, and may be an emerging avenue.

**Table 2:** KDM6 in cancer therapy.

Context	Agents	Mechanism	Type of Cancer	Function	Refs
Targeting KDM6	MC3324 Metformin	KDM1A and KDM6A inhibitor KDM6A inhibitor	Breast Cancer Bladder Cancer	Regulating drug resistance of hormone signaling Promoting a H3K27me3 enriched epigenome	(128, 129)
	GSK-J1 GSK-J4	KDM6A and KDM6B inhibitor KDM6A and KDM6B inhibitor	Head Neck Squamous Cell Carcinoma Acute Myeloid Leukemia Glioma Pleural Mesothelioma Prostate Cancer B-cell Lymphoma Ovarian Cancer	Synergistic inhibition of KDM6B and KDM1A by GSK-J1 and TCP *in vivo* Inhibiting cancer cell proliferation, and particularly exhibiting an inhibitory effect *in vivo* in PDX model of acute myeloid leukemia, breast cancer, T-cell acute lymphoblastic leukemia, colorectal cancer and osteosarcoma	(74, 78, 93, 99, 106, 130–137)
			Breast Cancer Gastric Cancer Neuroblastoma		
			T-cell Acute Lymphoblastic Leukemia Colorectal Cancer Osteosarcoma		
Exploiting KDM6 loss	GSK343/ GSK126	EZH2 inhibitors	Multiple Myeloma	KDM6A loss confers sensitivity to EZH2 inhibitors	(55)
	GSK503/ EPZ6438	EZH2 inhibitors	Bladder Cancer	KDM6A loss confers sensitivity to EZH2 inhibitors *in vivo*.	(138, 139)
	JQ1 Vorinostat	BET inhibitors HDAC inhibitor	Pancreatic Cancer Pancreatic Cancer	KDM6A loss confers sensitivity to BET inhibitors *in vivo* KDM6A loss confers sensitivity to HDAC inhibitor *in vivo*	(58, 140)
	Tocilizumab/ Propagermanium	Immune checkpoint inhibitors	Bladder Cancer	KDM6A loss confers sensitivity to combined inhibition of IL-6 and CCL2	(120)

KDM6A, a known tumor suppressor, is frequently inactivated in cancers (143). Given that the imbalance between KDM6A and EZH2 expression appears to sensitize many cancers to epigenetic inhibitors, resetting epigenetic balance could be useful in combination approaches (13). In multiple myeloma and bladder cancer, KDM6A loss confers sensitivity to inhibitors of the EZH2 methyltransferase, while KDM6A loss in pancreatic cancers sensitizes to bromodomain and extra-terminal (BET) and histone deacetylase (HDAC) inhibitors (**Table 2**) (55, 58, 138–140). In several cancers, KDM6B is downregulated and regarded as a tumor suppressor, suggesting there are no effects of KDM6 inhibitors in these cellular contexts (88–92). KDM6B loss conferring sensitivity to inhibitors of EZH2, BET and HDAC should be investigated in future. However, mechanistic details of how loss of KDM6 stimulates enhancer activation are lacking.

Because some epigenetic regulators have shown potent immunomodulatory activity, combination with immune checkpoint inhibitors (ICIs) could be promising (144). Notably, KDM6 demethylases regulates multiple innate and adaptive immune responses (145, 146). Furthermore, in bladder cancer KDM6A loss activates cytokine and chemokine pathways, and these cells are more sensitive to combined inhibition of IL-6 and CCL2 (**Table 2**) (120). A number of trials combining epigenetic inhibitors with ICIs are in progress, and need to be explored (147).

## Conclusions and Future Perspectives

Because functional roles of KDM6 subfamily were highly cell type-specific and pathologic context-dependent, it is essential to consider different cellular functions of KDM6 subfamily when targeting their highly related enzymatic domains. Therefore, developing a deeper understanding of differential roles and associations of KDM6 subfamily in cancer will provide precision therapeutic strategies. In addition, development of highly specific small-molecule inhibitors of KDM6 subfamily will be of utmost important in modern molecular oncology.

The regulation of KDM6 is complex and intriguing, and involves the coordinated control between cellular metabolism (intrinsic regulators) and tumor microenvironment (extrinsic stimuli). A better understanding of regulatory mechanisms of KDM6 demethylases in cancer progression will guide provide novel targeted therapeutic options for epigenetic inhibitors.

Due to potent immunomodulatory activities of KDM6, ICIs should be taken into consideration when targeting the KDM6 demethylases. Considering the discussed effects KDM6 exerts on both innate and adaptive immune cells, it is essential to understand how targeting the KDM6 demethylases would affect tumor immune microenvironment. Development of combining epigenetic inhibitors with ICIs will complement the existing arsenal of epigenetic drugs.

## Author Contributions

JC, SL, JZ, and JF performed paper searches and wrote the manuscript. CH, WS, and WW designed the study, revised the manuscript and made the decision to submit. All authors contributed to the article and approved the submitted version.

## Funding

This work was supported by National Natural Science Foundation of China (81802963, 81972261, 81901660), Zhejiang Provincial Natural Science Foundation of China (LY18H160046), Zhejiang Medical Science Foundation (2018KY531), Lin He’s New Medicine and Clinical Translation Academician Workstation Research Fund (18331215).

## Conflict of Interest

The authors declare that the research was conducted in the absence of any commercial or financial relationships that could be construed as a potential conflict of interest.

## Publisher’s Note

All claims expressed in this article are solely those of the authors and do not necessarily represent those of their affiliated organizations, or those of the publisher, the editors and the reviewers. Any product that may be evaluated in this article, or claim that may be made by its manufacturer, is not guaranteed or endorsed by the publisher.

## Glossary

AP-1: Activator protein 1
AR: Androgen receptor
ARNT: Aryl hydrocarbon nuclear translocator
BET: Bromodomain and extra-terminal
BMP4: Bone morphogenetic protein 4
CCL2: C-C chemokine ligand 2
CDK1: Cyclin-dependent kinase 1
C/EBPβ: CCAAT/enhancer-binding protein beta
EGF: Epidermal growth factor
EMT: Epithelial-mesenchymal transition
ER: Estrogen receptor
EZH2: Enhancer of zeste homolog 2
FGFR3: Fibroblast growth factor receptor 3
FH: Fumarase hydratase
FIH1: Factor-inhibiting hypoxia-inducible factor 1
HDAC: Histone deacetylase
HER2: Human epidermal growth factor receptor 2
HIF-1α: Hypoxia-inducible factor-1α
HRE: Hypoxia response element
H3K27: Histone H3 lysine 27
H3K27me2: H3K27 dimethylation
H3K27me3: H3K27 trimethylation
ICIs: Immune checkpoint inhibitors
IDH1 and 2: Isocitrate dehydrogenase 1 and 2
IKK: Inhibitor of nuclear factor-κ kinase
IL-6: Interleukin-6
IRF4: Interferon regulatory factor 4
JAKs: Janus kinases
JmjC: Jumonji C
JMJD3: Jumonji domain-containing protein D3
KDM: Lysine demethylase
LDHA: Lactate dehydrogenase A
LPS: Lipopolysaccharides
MAPK: Mitogen-activated protein kinase
NEFM: Neurofilament medium
NF-κB: Nuclear factor-κB
PHDs: Prolyl hydroxylases
PTEN: Phosphatase and tensin homolog deleted on chromosome ten
RAR: Retinoic acid receptor
SDH: Succinate dehydrogenase
STAT3: Signal transducer and activator of transcription 3
T-ALL: T-cell acute lymphoblastic leukemia
TAL1: T-cell acute leukemia protein 1
TCA: Tricarboxylic acid
TCGA: The Cancer Genome Atlas
TGF-β: Transforming growth factor-β
TLR4: Toll like receptor 4
TNFα: Tumor necrosis factor α
TPR: Tetratricopeptide repeat domain
TRAF6: TNF receptor associated factor 6
UC: Urothelial cancer
UTX: Ubiquitously transcribed tetratricopeptide repeat X
UTY: Ubiquitously transcribed tetratricopeptide repeat Y
1,25(OH) (2)D (3): 1α,25-dihydroxyvitamin D (3)
2-HG: 2-hydroxyglutarate
2-OG: 2-oxoglutarate
α-KG: α-ketoglutarate
